# Effectiveness of Functional Electrical Stimulation Assisted Locomotor Training on walking Outcomes Following Incomplete Spinal Cord Injury: Systematic Review and Meta-Analysis

**DOI:** 10.1177/15459683251395722

**Published:** 2025-12-08

**Authors:** Janelle Unger, Joshua C. Wiener, Prachi Patel, Usman Shakir, Janice J. Eng

**Affiliations:** 1 School of Physical Therapy, Faculty of Health Sciences, Western University, London, ON, Canada; 2 Gray Centre for Mobility and Activity, Parkwood Institute, St. Joseph’s Healthcare, London, ON, Canada; 3 Interdisciplinary Medical Sciences Program, Schulich School of Medicine & Dentistry, Western University, London, ON, Canada; 4 Department of Physical Therapy, Faculty of Medicine, University of British Columbia and Centre for Aging SMART, Vancouver, BC, Canada

**Keywords:** gait, spinal cord injuries, electric stimulation, walk test, systematic review

## Abstract

**Introduction:**

Functional electrical stimulation (FES) may enhance the impact of locomotor training on walking impairments following spinal cord injury (SCI).

**Objective:**

This systematic review (PROSPERO: CRD42023435210) evaluated the therapeutic effectiveness of FES-assisted locomotor training (FALT) on improving walking speed and endurance for individuals with motor incomplete SCI (iSCI).

**Methods:**

Databases (MEDLINE, EMBASE, CINAHL) were searched for interventional studies of FALT in iSCI that assessed the therapeutic effects on walking speed and/or endurance when the FES was not active. Study characteristics and findings were extracted, summarized, and narratively synthesized. Risk of bias was assessed using the Cochrane tools for interventional studies. Random effects meta-analyses were conducted to generate standardized pooled effect sizes for both outcomes.

**Results:**

Thirteen studies were identified: 4 randomized controlled trials (RCTs) and 9 pre-post tests. RCTs scored low (*n* = 1 study), intermediate (*n* = 1), and high (*n* = 2) on the RoB2, and all pre-post tests studies (*n* = 9) scored high on the ROBINS-I. Meta-analyses of 3 RCTs found that treadmill-based FALT was associated with a small, non-significant effect on walking speed (*n* = 76 participants; Hedge’s *g*: −0.01; 95% CI: −0.46, 0.43; *P* = .96) and a small, non-significant effect on walking endurance (*n* = 71; Hedge’s *g*: 0.20; 95% CI: −0.25, 0.65; *P* = .39) when compared to control conditions.

**Discussion:**

This review did not find evidence that FALT improves walking speed or endurance for people with iSCI relative to other types of locomotor training. Future trials of FALT for SCI should aim to better standardize and report training dose and stimulation parameters to improve comparability.

## Introduction

Gait impairments are common following a spinal cord injury (SCI),^1^ and improved walking is often identified as a priority in this population.^2^ Individuals with motor incomplete SCI—in which some motor function is preserved below the level of the lesion—have the best prognosis to regain walking function after injury,^3^ due to greater intact motor pathways in both spinal and supraspinal networks.^4^ In the past 2 decades, SCI rehabilitation has shifted focus from using compensatory strategies, such as providing assistive devices, to harnessing neuroplasticity, which can help recover walking function by strengthening existing and creating new neural connections.^5^ Exercise-induced neuroplasticity is the most effective treatment for SCI-related impairments through neurotrophic and synaptic changes as well as axonal sprouting and regeneration.^6^ This activity-dependent plasticity is the basis for Activity-Based Therapy (ABT), which requires both task-specific training and neuromuscular activation below the level of the lesion to drive changes within the nervous and muscular systems in order to improve function.^7^

While many types of rehabilitation therapies exist for improving walking outcomes for individuals with SCI,^8^ locomotor training is the most common intervention used for this purpose,^9^ due to its specificity. Locomotor training can be performed overground or on a treadmill, and it can incorporate various technologies such as body-weight support or robotic assistance. The effects of these types of locomotor training are inconclusive, where many quasi-experimental studies show improvements,^8^ but such findings are not supported by experimental studies or systematic reviews.^10^ For example, a recent network meta-analysis found no differences between overground training with body-weight support, body-weight supported treadmill training (BWSTT), and robotic-assisted locomotor training (RALT) on walking ability as measured using several outcomes, including speed and endurance.^11^ Similarly, another recent network meta-analysis comparing 2 types of RALT—exoskeleton and Lokomat—found similar results between the 2 methods for improving walking function.^12^ These findings align with an earlier review that reported only minor differences between locomotor training approaches, without any one method showing superiority.^13^ Continued evaluation of innovative locomotor training approaches is necessary.

Neuromodulation is a rehabilitation approach that involves either direct stimulation to the central nervous system (ie, brain or spinal cord) or stimulation to the peripheral nerves and muscles to augment function.^14^ Neuromodulation techniques using electrical stimulation are increasingly used as part of SCI rehabilitation,^9^ and preliminary research on combining these techniques with locomotor training shows potential in improving walking function. Functional electrical stimulation (FES) is a type of peripheral stimulation where the electrical stimulus is placed over key muscles or peripheral nerves to assist with functional or purposeful movements such as walking.^15^ FES was initially used as a neuroprosthesis to improve user function only while wearing the device, thereby producing an orthotic effect. However, when paired with task-specific locomotor training, FES may enhance muscle activation and engagement during training. Some studies have reported that functional improvements persist beyond the intervention period, which suggests a therapeutic effect of the combined intervention.^15^ Given that FES is an accessible and affordable neuromodulation technique, as well as the only one widely used in usual care, it is critical to establish whether its integration into locomotor training provides added therapeutic benefits.^14,15^

Previous systematic reviews on the therapeutic effectiveness of combining FES with locomotor training—hereby referred to as FES-assisted locomotor training (FALT)—are conflicting and based on limited research. A systematic review from 2007 of 1 randomized controlled trial (RCT) and 3 pre-post studies found that FALT resulted in improved functional ambulation in individuals with motor incomplete SCI, as measured by both speed and endurance.^8^ These findings were shown to persist after the stimulation ceased, which suggests that neuroplastic changes occurred.^8^ However, a 2010 Cochrane review of 2 RCTs did not find statistically significant effects of FES-assisted BWSTT on speed or endurance, although only the data on walking speed was able to be pooled.^10^ Since the publication of these reviews, additional literature on FALT has been published, including additional RCTs, which warrants an updated systematic review and meta-analysis.

Therefore, the objective of this systematic review and meta-analysis was to evaluate the therapeutic effectiveness of FALT on improving walking speed and endurance for people with motor incomplete SCI.

## Methods

This review was composed in accordance with the Preferred Reporting Items for Systematic Reviews and Meta-Analyses (PRISMA) guidelines, as updated in 2020.^16,17^ The protocol for this review was registered on PROSPERO (CRD42023435210). The review question and search strategy were developed using PICO criteria^18^ (Table 1).

**Table 1.** Research Question.

Criterion	
Population	Adults with motor incomplete spinal cord injury
Intervention	Functional electrical stimulation (FES)-assisted locomotor training, either on a treadmill or overground
Comparison	Any type of control group not receiving FES-assisted locomotor training
Outcomes	Walking speed, as assessed by a measured walking test, such as the 10-Metre Walk Test (10MWT) Walking endurance, as assessed by a timed walking test, such as the 6-Minute Walk Test (6MWT)

### Search Strategy

The following databases were searched from inception to July 2025: MEDLINE (Ovid), EMBASE (Ovid), and CINAHL (Ebsco). Search terms were organized into 3 concepts: (1) spinal cord injury (eg, spinal cord injuries, paraplegia, tetraplegia); (2) FES (eg, electrical stimulation, electrostimulation); and (3) walking outcomes (eg, gait, walking, speed, endurance). These terms were searched using database-specific subject headings and/or keywords and then linked using Boolean operators. The complete search strategies are available in the Supplemental Appendix.

### Eligibility Criteria

Studies were eligible for inclusion if: (1) the study design was experimental (ie, RCT; parallel or crossover) or quasi-experimental (ie, pre-post test; controlled or uncontrolled); (2) the participants were adults with motor incomplete SCI at any spinal level; (3) the intervention was any type of FALT program, conducted with or without body-weight support, and completed either on a treadmill or overground; and (4) the outcomes included walking speed (as assessed by the 10-Metre Walk Test [10MWT] or another measured walking test) and/or walking endurance (as assessed by the 6-Minute Walk Test [6MWT] or another timed walking test) performed overground and/or on a treadmill without FES (ie, therapeutic effect). Studies were excluded if they: (1) reported on ineligible populations without stratifying results (eg, pediatric (<18 years old), complete SCI, stroke); (2) evaluated FES and locomotor training as 2 separate interventions, or other types of FES training (eg, cycling, rowing); (3) only assessed walking speed and/or endurance performed with FES (ie, orthotic effect and not the therapeutic effects when the FES is no longer used); and (4) were not published in English as a full-text, peer-reviewed, primary research article (eg, abstract, review).

### Study Screening

Search results were imported into Covidence systematic review software (Veritas Health Innovation, 2023, Melbourne, Australia) for deduplication and screening. Two independent reviewers (JU & JCW) screened the articles at Level 1 (ie, title and abstract) and Level 2 (ie, full text) for inclusion and resolved disagreement through consensus. Backward and forward citation searching of included articles and other relevant reviews was conducted to identify studies that may have been missed by the database searches.

### Study Appraisal

Risk of bias for included studies was assessed using the Cochrane tools for experimental studies (risk of bias tool for randomized trials Version 2 [RoB2])^19^ and quasi-experimental studies (risk of bias in non-randomized studies—for interventions [ROBINS-I Version 2]).^20^ Two independent reviewers (JU & JCW) assessed the studies and resolved disagreement through consensus. Each study was rated as having an overall low, moderate, or high risk of bias. As well, RCTs were examined using scores from the Physiotherapy Evidence Database (PEDro).^21^ Each study was classified as poor (0-3), fair (4-5), good (6-8), or excellent (9-10) based on the total score.^22^

### Data Extraction

Study data were extracted into a pre-established data extraction tool by 2 independent reviewers (PP & US), with disagreements resolved by a third reviewer (JCW). Extracted data included study details (eg, year, location, design), participant characteristics (eg, age, sex, duration of injury, level of injury, severity of injury), intervention details (eg, type, frequency, intensity, duration), outcome measures (ie, speed and/or endurance), and study findings (ie, data from text, tables, and figures).

### Data Synthesis

The characteristics and findings of all included studies were synthesized into summary tables and summarized within the text. Meta-analyses were conducted using reported data from articles of RCTs. Standardized pooled effect sizes (Hedge’s *g*) were calculated using random effects models based on the sample sizes, standard errors, and mean changes in the treatment and control groups from each RCT.^23^ In cases where these specific values were not reported, pre- and post-intervention mean and standard deviation were used to calculate mean changes with standard errors, and confidence intervals were used to calculate standard errors.^24^ All analyses were conducted in Stata 18.0 (StataCorp, 2023, College Station, Texas, USA) using the *meta* command suite.

Effect sizes were interpreted as small (0.2-0.5), medium (0.5-0.8), or large (≥0.8).^25^ Statistical heterogeneity was assessed using the *I*^2^ statistic and Cochrane’s *Q* test. The *I*^2^ statistic was used to interpret heterogeneity as low (>25%), moderate (>50%), or high (>75%).^26,27^ The Cochrane’s *Q* test was used to determine statistically significant heterogeneity (*P* < .01).^26,27^ A funnel plot was used to examine the potential for publication bias.^28^

## Results

### Study Selection

**Figure 1.**
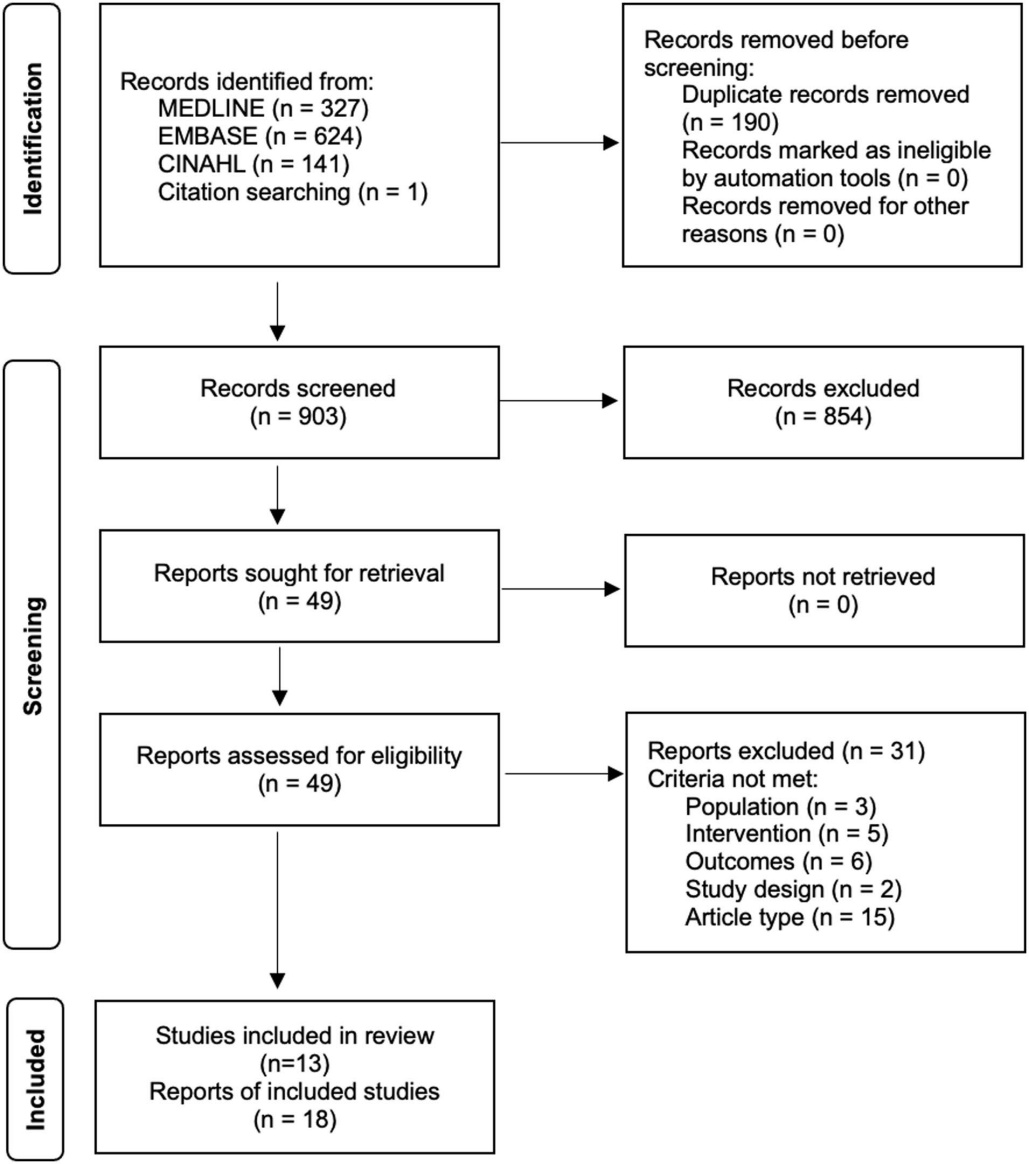
Preferred Reporting Items for Systematic Reviews and Meta-Analyses (PRISMA) 2020 flow diagram for new systematic reviews.

An outline of the screening process is presented in Figure 1. A total of 903 and 49 articles were screened at Level 1 and Level 2, respectively. Articles were excluded due to not meeting the inclusion criteria for population (*n* = 3), intervention (*n* = 5), outcomes (*n* = 6), and study design (*n* = 2), as well as not being a full-text article (*n* = 15). Ultimately, 18 articles were included in the review, representing 13 unique studies: 4 RCTs and 9 single-group, pre-post studies.

### Study Details

#### Experimental Studies

**Figure 2.**
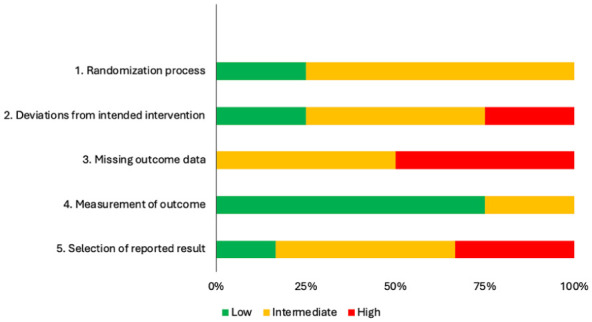
Summary of risk of bias assessment of randomized controlled trials using the RoB 2 tool.

The details of the 4 RCTs—3 of which were each published across 2 articles—are summarized in Table 2. Three RCTs used a parallel design with 2 or more groups (eg, treatment and control),^29-34^ and 1 used a crossover design with 2 groups whose order of intervention was randomized (ie, treatment then control, or control then treatment).^35^ On the RoB2, RCTs were scored as having low (*n* = 1),^29,30^ intermediate (*n* = 1),^31,32^ or high (*n* = 2)^33-35^ risk of bias. The main concerns for risk of bias were missing outcome data, selection of reported result, and deviations from intended intervention (Figure 2). According to the PEDro database, scores ranged from 4 (*fair*) to 7 (*good*); 2 RCTs had conflicting scores between their 2 respective publications.^29-32^

**Table 2.** Details of Randomized Controlled Trials.

Study	ROB PEDro	Participants	Interventions	Findings
Postans et al^35^	High 4	Sample: *n*_Start_ = 14, *n*_End_ = 10 Age: 41.8 ± 14.7 years Sex: Male = 12, Female = 2 Duration: 12.2 ± 5.9 weeks Level: C4-T9 Severity: AIS C = 8, *D* = 6	*Intervention* LT: Treadmill, ≤40% body weight support FES: Parameters not reported Dose: 25 minutes/day, 5 days/week, 4 weeks *Control* Standard physiotherapy care; 5 days/week, 4 weeks *Groups* Group 1: Control → Intervention (n = 5) Group 2: Intervention → Control (n = 5) *(Note: Crossover trial with order randomized.)*	***Speed*** (6-Metre Walk Test) Greater mean increase overground after Intervention than Control for Group 1 (0.23 m/second, 95% CI 0.13-0.33, *P* = .004 vs .15 m/second, 95% CI −0.04-0.34, *P* = .096) but not Group 2 (0.17 m/second, 95% CI −0.15-0.45, *P* = .160 vs .22 m/second, 95% CI −0.05-0.37, *P* = .019). (*Note: No between-group comparison conducted.*) *Endurance* (6-Minute Walk Test) Greater mean increase overground after Intervention than Control for Group 1 (72.2 m, 95% CI 39.8-104.6, *P* = .003 vs 38.4 m, 95% CI 1.8-75.0, *P* = .044) but not Group 2 (63.8 m, 95% CI −10.2-137.9, *P* = .075 vs 60.1 m, 95% CI 9.2-110.9, *P* = .030). (*Note: No between-group comparison conducted.*)
Field-Fote and Roach^32^/Kressler et al^31^	Inter-mediate 6/5	*Intervention 1* Sample: n_Start_ = 18, n_End_ = 15; Age: 42.2 ± 15.7 years Sex: Male = 11, Female = 4 *Intervention 2* Sample: n_Start_ = 22, n_End_ = 18 Age: 38.5 ± 12.7 years Sex: Male = 14, Female = 4 ***Intervention 3*** Sample: n_Start_ = 15, n_End_ = 14 Age: 45.0 ± 8.0 years Sex: Male = 12, Female = 2 *Intervention 4* Sample: n_Start_ = 19, n_End_ = 17 Age: 39.3 ± 14.6 years Sex: Male = 14, Female = 3 **All groups** Duration: ≥1 year Level: T10 or higher Severity: AIS C or D	*Intervention 1* LT: Overground, ≤30% body weight support FES: Parameters not reported Dose: 5 days/week, 12 weeks *Intervention 2* LT: Treadmill, ≤30% BWS FES: Parameters not reported Dose: 5 days/week, 12 weeks *Intervention 3* LT: Treadmill, ≤30% BWS, Robotic assistance Dose: 5 days/week, 12 weeks *Intervention 4* LT: Treadmill, ≤30% BWS, Manual assistance Dose: 5 days/week, 12 weeks	*Speed* (10-Metre Walk Test) Significant increase overground after Intervention 1 (0.09 ± 0.11 m/second, ES = 0.43, *P* < .05), Intervention 2 (0.05 ± 0.09 m/second, ES = 0.28, *P* < .05), and Intervention 4 (0.04 ± 0.07 m/second, ES = 0.28, *P* < .05), but not Intervention 3 (0.01 ± 0.05 m/second, ES = 0.10, *P* > .05). No significant difference between groups over time (*P* = .0930). *Endurance* (2-Minute Walk Test) Significant increase overground after Intervention 1 (14.2 ± 15.2 m, ES = 0.40, *P* < .05) and Intervention 2 (3.8 ± 6.3 m, ES = 0.16, *P* < .05), but not Intervention 3 (1.2 ± 3.2 m, ES = 0.17, *P* > .05) or Intervention 4 (0.8 ± 7.7 m, ES = 0.04, *P* > .05). Significant difference between groups over time (*P* = .0004), with greater increase after Intervention 1 than Interventions 2 to 4 (*P* < .01).
Giangregorio et al^29^/Kapadia et al^30^	Low 5/7	*Intervention* Sample: n_Start_ = 17, n_End_ = 16 Age: 56.6 ± 14.0 years Sex: Male = 14, Female = 3 Duration: 8.75 ± 9.7 years Level: C2-T12 Severity: AIS C or D *Control* Sample: n_Start_ = 17, n_End_ = 11 Age: 54.4 ± 16.5 years Sex: Male = 12, Female = 5 Duration: 10.3 ± 11.1 years Level: C2-T12 Severity: AIS C or D	*Intervention* LT: Treadmill, Body weight support FES: 20-50 Hz, 0-300 µs, 8-125 mA Dose: 45 minutes/day, 3 days/week, 16 weeks *Control* LT with aerobic and resistance training; 45 minutes/day, 3 days/week, 16 weeks	*Speed* (10-Metre Walk Test) Mean increase overground after Intervention (0.05 ± 0.08 m/second) and Control (0.14 ± 0.46 m/second). No significant effect of time (*P* = .084), group (*P* = .0829), or group over time (*P* = .195). *Endurance* (6-Minute Walk Test) Mean increase overground after Intervention (29.2 ± 60.8 m) and Control (51.5 ± 36.2 m). Significant effect of time (*P* = .002), but not group (*P* = .096) or group over time (*P* = .732).
Jones et al^33^/Jones et al^34^	High 5	*Intervention* Sample: n_Start_ = 26, n_End_ = 20 Age: 42.2 ± 13.0 years Sex: Male = 19, Female = 1 Duration: 77.8 ± 122.5 months Level: Para = 5, Tetra = 15 Severity: AIS C = 7, D = 13 *Control* Sample: n_Start_ = 22, n_End_ = 21 Age: 34.1 ± 12.0 years Sex: Male = 11, Female = 10 Duration: 75.3 ± 88.3 months Level: Para = 5, Tetra = 16 Severity: AIS C = 11, D = 10	*Intervention* Activity-based therapy (locomotor training, resistance training, developmental sequencing) LT: Overground and/or treadmill, Manual and/or robotic assistance, Body weight support FES: Parameters not reported Dose: 3 hour/day, 3 days/week, 24 weeks *Control* No intervention (waitlist); 24 weeks	*Speed* (10-Metre Walk Test) Significantly greater mean increase after Intervention (0.096 ± 0.140 m/second) than Control (0.027 ± 0.104) over time (*P* = .036). *Endurance* (6-Minute Walk Test) Significantly greater mean increase after Intervention (35.97 ± 48.15 m) than Control (3.00 ± 25.51 m) over time (*P* = .002).

Abbreviations: AIS, American Spinal Injury Association Impairment Scale; BWS, body weight support; CI, confidence interval; ES, effect size; FES, functional electrical stimulation; LT, locomotor training; PEDro, Physiotherapy Evidence Database; ROB, risk of bias.

The initial sample sizes ranged from 14 to 74 participants, with a pooled initial sample size of 170. The final sample sizes ranged from 10 to 64 participants, due to attrition across all RCTs (range: 4-10), with a pooled final sample size of 142. The study samples were predominantly male, with a reported 119 male and 34 female participants. The mean age of the samples ranged from 38.5 ± 13.1 to 55.5 ± 15.1 years, with an overall mean age of 43.2 years. The mean duration of injury ranged from 12.2 ± 5.9 weeks to 9.5 ± 10.3 years, with an overall mean duration of 6.6 years; 1 study reported that all participants had injuries for ≥1 year but did not provide specifics. Participants’ level of injuries spanned from the cervical to thoracic regions (range: C2-T12); 1 RCT did not report level of injuries.^33,34^ Participants’ impairments were classified on the American Spinal Injury Association (ASIA) Impairment Scale (AIS) as Grade C or D.

The type of locomotor training across RCTs included supervised treadmill training (*n* = 4)^29-35^ and overground training (*n* = 3),^31-35^ which was augmented with body weight support (*n* = 4),^29-35^ robotic assistance (*n* = 2),^31-34^ and/or manual assistance (*n* = 2).^31-34^ Three RCTs compared FALT to a specific control condition: aerobic and resistance training (*n* = 1),^29,30^ standard physiotherapy care (*n* = 1),^35^ and no intervention (*n* = 1).^33,34^ One RCT compared 4 locomotor training interventions, with no specified control condition: FALT overground, FALT on a treadmill, with manual assistance on a treadmill, and with robotic assistance on a treadmill.^31,32^ Three RCTs utilized an active control,^29-32,35^ while 1 used an inactive/waitlist control.^33,34^ The dose of training varied substantially across RCTs: from 25 minutes to 3 hours in daily duration; from 3 to 5 days in weekly frequency; and from 4 to 24 weeks in total intervention duration. The FES parameters were only reported in 1 RCT: 20 to 50 Hz in pulse frequency, 0 to 300 µs in pulse duration, and 8 to 125 mA in pulse amplitude.^29,30^

For the meta-analysis, only the 3 RCTs with an active control group were included to ensure comparability of effects across the control groups.^29-32,35^ For the RCT with a crossover design, the 2 groups were pooled according to intervention (ie, treatment and control). For the RCT with 4 interventions,^31,32^ treadmill-based FALT (ie, treatment) was compared to treadmill-based, manually-assisted locomotor training (ie, control) to provide the most direct comparison.

#### Quasi-Experimental Studies

**Figure 3.**
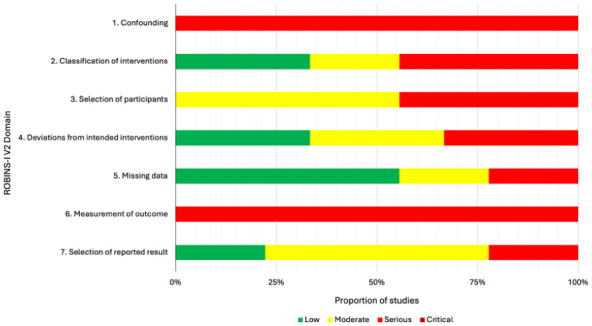
Summary of risk of bias assessment of quasi-experimental studies using the ROBINS-I tool.

The details of the 9 pre-post studies—2 of which were each published across 2 articles—are summarized in Table 3.^36-46^ On the ROBINS-I, all of these studies were scored as having a serious risk of bias.^36-46^ The main concerns for risk of bias were due to confounding factors and measurement of outcomes (Figure 3).

**Table 3.** Details of Quasi-Experimental Studies.

Study	Region	Design	ROB	Participants	Interventions	Findings
Granat et al^36^	UK	Pre-post test	Serious	Sample: *n* = 6 Age: 31.5 ± 7.1 years Sex: Male = 3, female = 3 Duration: 7.3 ± 5.9 years Level: C3-L1 Severity: Frankel C = 3, D = 3	LT: Overground walking in community FES: 25 Hz, 300 µs, 20-100 mA Dose: ≥30 minutes/day, 5 days/week, ≥12 weeks	*Speed* No significant change overground. *(Note: Values not provided.)*
Wieler et al^37^	Canada	Pre-post test	Serious	Sample: *n* = 31 Age: 36.0 ± 2.0 years Sex: Not reported Duration: 6.0 ± 1.0 years Level: C1-T12 Severity: Not reported	LT: Overground walking in community FES: Parameters not reported Dose: Daily, ~1 year	*Speed* Significant mean increase overground (55%, P < 0.01).
Ladouceur and Barbeau^38^/Ladouceur and Barbeau^45^	Canada	Pre-post test	Serious	Sample: *n* = 14 Age: 33.0 ± 6.1 years Sex: Not reported Duration: 6.8 ± 4.6 years Level: C3-L1 Severity: AIS C = 5, D = 9	LT: Overground walking in community FES: 10-30 Hz, 50-500 µs, 0-150 mA, 0-100 V Dose: Daily, ~1 year	*Speed* Significant mean increase overground (0.01 ± 0.18 m/second, P < .01).
Field-Fote^39^/Field-Fote and Tepavac^40^	USA	Pre-post test	Serious	Sample: *n* = 19 Age: 31.7 ± 9.4 years Sex: Male = 13, female = 6 Duration: 69.9 ± 47.6 months Level: C5-T7 Severity: AIS C	LT: Treadmill, ≤30% body weight support FES: 1 ms/p, 50 p/second, 60-100 V Dose: ≤90 minutes/day, 3 days/week, 12 weeks	*Speed* Significant mean increase overground (0.09 ± 0.81 m/second, P < .001) and on treadmill (0.26 ± 0.23 m/second, P < .0001). *Endurance* Significant mean increase on treadmill (150.0 ± 162.4 m, P < .00001).
Hesse et al^41^	Germany	Pre-post test	Serious	Sample: *n* = 4 Age: 50.3 ± 8.3 years Sex: Male = 3, female = 1 Duration: 10.0 ± 6.3 months Level: C5-L2 Severity: AIS C = 1, D = 3	LT: Gait trainer, 10-35% body weight support FES: 20 Hz, 0.2 ms, 0-80 mA Dose: 20-25 minutes/day, 5 days/week, 5 weeks	*Speed* Mean increase overground (0.09 m/second). *(Note: No significance test conducted.)* *Endurance* Mean increase on treadmill (150 m). *(Note: no significance test conducted.)*
Thrasher et al^42^	Canada	Pre-post test	Serious	Sample: *n* = 5A ge: 49.0 ± 20.3 years Sex: Male = 2, female = 3 Duration: 9.0 ± 8.6 years Level: C5-T12 Severity: AIS C = 2, D = 2, NA = 1	LT: Treadmill and overground FES: 35 Hz, 0-300 µs, 18-110 mA Dose: 15-30 minutes/day, 2-5 days/week, 12-18 weeks	*Speed* (2-Metre Walk Test) Significant increase in 4/5 participants overground. (*Note: Values not reported; No within-group comparison conducted.*)
Sharif et al^43^	Canada	Pre-post test	Serious	Sample: *n* = 6 Age: 60.5 ± 13.2 years Sex: Male = 3, female = 3 Duration: 9.3 ± 12.0 years Level: C4-L3 Severity: AIS D	LT: Gait trainer, 15-55% body weight support FES: 35 Hz, 0-300 µs, 5-60 mA Dose: 25-50 minutes/day, 3 days/week, 12 weeks	*Speed* (10-Metre Walk Test) Non-significant mean increase overground (0.21 ± 0.64 m/second, ES = 0.38, *P* = .08). *Endurance* (6-Minute Walk Test) Significant mean increase overground (73.7 ± 217.0 m, ES = 0.48, *P* = .03).
Street and Singleton^44^	UK	Pre-post test	Serious	Sample: *n*_Start_ = 35, n_End_ = 24 Age: 53.0 ± 15.0 years Sex: Not reported Duration: 9 (0.4-39) years Level: T12 or higher Severity: AIS C or D	LT: Overground walking in community FES: 40 Hz, 0-360 µs, 0-100 mA Dose: Daily, 6 months	*Speed* (10-Metre Walk Test) Significant mean increase overground (0.09, 95% CI 0.02-0.16, *P* = .025).
Berkelmans et al^46^	Netherlands	Pre-post test	Serious	Sample: *n* = 5 Age: 40.8 ± 12.28 years Sex: Male = 3, female = 2 Duration: 7.0 ± 8.49 years Level: C5-T12 Severity: AIS C	LT: Treadmill FES: 50 Hz, 1-300 µs, ~50 mA Dose: 30 minutes/day, 2 days/week, 10 weeks	*Speed* (10-Metre Walk Test) Increase in 4/5 participants overground. (*Note: Values not reported; No within-group comparison conducted.*)

Abbreviations: AIS, American Spinal Injury Association Impairment Scale; CI, confidence interval; ES, effect size; FES, functional electrical stimulation; LT, locomotor training; NA, not applicable; ROB, risk of bias.

The sample sizes ranged from 4 to 35 participants, with a pooled sample size of 131; the largest study reported attrition of 11 participants.^44^ Sex was not reported in 3 studies with the largest sample sizes^37,38,44,45^; the remaining 6 studies reported 30 male and 21 female participants. The mean age of the samples ranged from 29.5 ± 7.8 to 60.5 ± 13.2 years, with an overall mean age of 41.9 years. The mean duration of injury ranged from 0.83 ± 0.53 to 9.3 ± 12.0 years, with an overall mean duration of 7.1 years. Participants’ level of injuries spanned from the cervical to lumbar regions and impairments were classified on the AIS as Grade C or D; 1 study did not report on participants’ specific classification.^37^

The type of locomotor training included overground walking in the community (indoor and outdoor) (*n* = 4),^36-38,44,45^ supervised training on a treadmill or gait trainer with body weight support (*n* = 3),^39-41,43^ supervised training on a treadmill without body weight support (*n* = 1),^46^ and supervised training both on a treadmill and overground (*n* = 1).^42^ The dose of training varied substantially across studies: from 20 to 25 minutes to several hours in daily duration; from 2 to 7 days in weekly frequency; and from 5 weeks to 1 year in total intervention duration. The FES parameters varied across studies in terms of the maximum pulse frequency (20-50 Hz), duration (1-500 µs), and amplitude (50-150 mA); 1 study did not report FES parameters.^37^

### Study Findings

The included studies measured the therapeutic effects of a multi-week FALT program on the walking measures that is the sustained improvements when the FES was removed or turned off.

#### Walking Speed

Walking speed was evaluated in all 4 RCTs using a measured walking test over either 6 m (*n* = 1)^35^ or 10 m (*n* = 3)^29-35^ overground. Three RCTs reported significant increases in speed following the intervention,^31-35^ but only 2 studies conducted between-group comparisons.^31-34^ Of these 2 studies, 1 RCT with high risk of bias found that the increase in speed over time was significantly greater following 24 weeks of ABT with FALT than a waitlist control,^33,34^ whereas the other RCT with intermediate risk of bias did not find any significant differences between FALT (overground or on treadmill) and 2 other locomotor training interventions (with manual or robotic assistance) after 5 weeks.^31,32^ Moreover, 1 RCT with low risk of bias reported that there were no significant effects of treadmill-based FALT on speed relative to aerobic-resistance training after 16 weeks of the intervention.^29,30^

**Figure 4.**
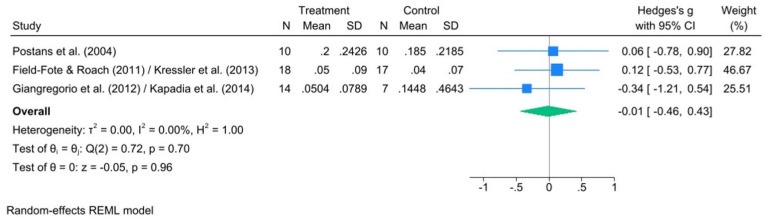
Forest plot comparing the effect of FES-assisted locomotor training versus active control on walking speed as assessed by a measured walking test, such as the 10-Metre Walk Test (10MWT).

Based on the meta-analysis of the 3 RCTs, there was a small, non-significant effect of treadmill FALT interventions (*n* = 42) relative to control conditions (*n* = 34) on walking speed (Hedge’s *g*: −0.01; 95% CI: −0.46, 0.43; *P* = .96) with non-significant heterogeneity between studies (*I*^2^ = 0%; *Q* = 0.72, *df* = 2, *P* = .70) (Figure 4).

All 9 quasi-experimental studies evaluated overground walking speed,^36-46^ with 4 studies using formal measured walk tests of either 2 m (*n* = 1)^42^ or 10 m (*n* = 3).^43,44,46^. Six pre-post studies reported significant increases in speed following FALT, which was either 6 to 12 months of community-based FALT (*n* = 3)^37,38,44,45^ or 10 to 18 weeks of supervised FALT (*n* = 2).^39,40,42,46^ These increases were demonstrated either through the mean change^37-40,44,45^ or in the majority of participants.^42,46^ One pre-post study reported an increase in speed after 5 weeks of supervised FALT but did not conduct statistical testing.^41^ Moreover, 2 pre-post studies reported increases in speed following 12 weeks of supervised FALT or community-based FALT, although these findings were not statistically significant.^36,43^

#### Walking Endurance

Walking endurance was evaluated in all 4 RCTs using a timed walking test completed over 2 minutes (*n* = 2),^31,32^ 4 minutes (*n* = 1),^29,30^ and/or 6 minutes (*n* = 3).^29,30,33-35^ All 4 RCTs reported statistically significant increases in endurance following FALT, but only 3 conducted between-group comparisons.^29-34^ Two RCTs found that the increase in endurance over time was significantly greater following 12 weeks of overground FALT or 24 weeks of ABT with FALT than control conditions.^31-34^ However, 1 RCT with low risk of bias reported that there was no significant effect of treadmill-based FALT on endurance relative to aerobic-resistance training after 16 weeks of the intervention.^29,30^

**Figure 5.**
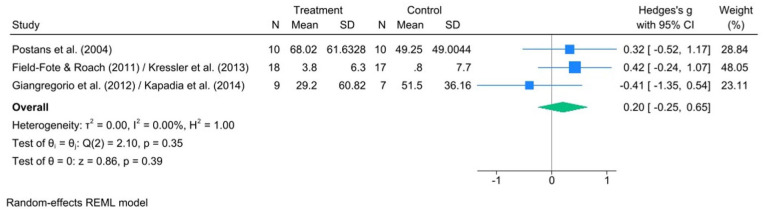
Forest plot comparing the effect of FES-assisted locomotor training versus active control on walking endurance as assessed by a timed walking test, such as the 6-Minute Walk Test (6MWT).

Based on the meta-analysis of the 3 RCTs, there was a small, non-significant effect of treadmill FALT interventions (*n* = 37) relative to control conditions (*n* = 34) on walking endurance (Hedge’s *g*: 0.20; 95% CI: −0.25, 0.65; *P* = .39) with non-significant heterogeneity between studies (*I*^2^ = 0%; *Q* = 2.10, *df* = 2, *P* = .35) (Figure 5).

Only 3 pre-post studies examined the effects of FALT on walking endurance, using the 6MWT (*n* = 1)^43^ or distance walked on a treadmill (*n* = 2).^39-41^ All 3 studies reported increases in the total distance walked following 5 to 12 weeks of supervised FALT,^39-41,43^ although only 2 studies confirmed these improvements with statistical testing.^39,40,43^

## Discussion

### Interpretation

The findings of this systematic review suggest that FALT may improve walking speed and endurance for people with motor incomplete SCI. When compared to other forms of locomotor training, however, there are no significant differences in walking speed or endurance outcomes. These findings are consistent with previous literature, where an early review found positive effects of FALT in a series of pre-post studies,^8^ and a later Cochrane review of 2 RCTs found no benefit of FALT compared to other types of locomotor training on walking speed and endurance.^10^ While the number of trials had increased since these reviews,^8,10^ studies continue to have small sample sizes and high risk of bias.

Based on the results of this meta-analysis, although non-significant, there were larger effects of FALT on walking endurance compared to walking speed, findings that were reflected in the pre-post studies that performed statistical analyses. Assessments of walking endurance may be more relevant than those for walking speed, as they represent how well someone can move throughout their community compared to walking for only brief periods. It has been suggested that walking speed may be too specific an outcome to assess efficacy of locomotor training. For instance, a systematic review of RALT did not find a significant increase in walking speed despite other gait parameters showing improvements.^47^ Endurance has been identified as a key component of community ambulation and has shown correlation with participation outcomes.^48^ Two of the RCTs^33-35^ in this review found that participants in the intervention groups experienced changes in their 6MWT scores beyond the minimal clinically important difference (MCID) of 36 m,^49^ while the participants in the control groups did not, which suggests these improvements hold clinical meaning. Studies that found significant effects of FALT on walking endurance implemented training at a frequency of 3 to 5 times per week for a period of 4 to 12 weeks, resulting in a total of approximately 17 to 72 sessions.^32-35,39,40,43^ Only one study reassessed participants at a longer term follow-up (2 and 8 months after the intervention ended) and reported no significant effects,^29,30^ so it is unclear if improvements in walking endurance can be maintained following treatment cessation using these protocols.

The explanations for the lack of significant effects on walking speed and endurance in this study are likely due to small sample sizes within the included studies as well as large variability within and across the study samples. These issues are intertwined, and they have been previously cited in other exercise-related research in this population.^50^ The number of individuals with SCI is low compared to other neurological impairments, such as stroke, leading to a small subset of the population eligible to participate in trials. Researchers often attempt to counteract this issue by using broad inclusion criteria to avoid further limiting their sample size and to maintain statistical power in the study.^50^ By widening inclusion criteria, more heterogeneity is introduced into the sample, which can reduce the likelihood of detecting statistically significant and clinically meaningful effects.^50^ However, homogeneity is difficult to attain even with strict inclusion criteria, due to the high amounts of variability that exist between individuals with the same injury classification or diagnosis.^50^ One possible solution to the issue of small samples sizes in this population is to conduct meta-analyses once several small RCTs have been published. Pooling data from several studies will ensure adequate study power to increase confidence in findings, although some caution is still warranted as small trials often show larger effect sizes and sensitivity analyses are recommended.^51^ This approach will require that researchers are using standardized outcomes and reporting study methods and findings in detail consistently.

Other issues with the included studies were the lack of description regarding the FES parameters and inconsistent treatment dosages, which limit the reproducibility of the research as well as the clinical application. It is possible the included studies used varying stimulation parameters, but it is unclear if there was heterogeneity in their approaches due to lack of reporting. Given the importance of training duration and intensity for neuroplasticity, future studies should prioritize standardized training doses and stimulation parameters, as well as explore whether a minimum threshold of practice is necessary for achieving lasting changes in walking function. Moreover, long-term follow-up assessments would help determine whether the improvements observed in some studies are sustainable after the cessation of training. These practices allow for better interpretation of the mechanisms underlying FALT effectiveness.

Lastly, the outcome measures selected could also contribute to heterogeneity within the research. While walking speed was consistently measured with a timed walking test and walking endurance with a measured distance over a set time, there was variability in the exact distances and times used for speed and endurance tests, respectively. An international survey identified the 10MWT and 6MWT as the most common standardized outcome measures used to assess walking function following SCI,^52^ and these tests are part of the standardized assessment for functional ambulators in the Standing and Walking Assessment Tool used in SCI rehabilitation across Canada.^53^ In the future, researchers should use the 10MWT for speed and the 6MWT for endurance to facilitate future data pooling and better align with clinical practice.

Risk of bias remains a concern in this research area, as indicated by only one study in this review with a low risk of bias. This level of risk decreases confidence in the findings and makes interpretation difficult, but it is likely an inevitable occurrence in this field. A recent scoping review found several methodological and reporting issues in rehabilitation research, including inappropriate study designs, intervention-related challenges, and lack of blinding.^54^ Although there have been many calls for increased high quality RCTs in SCI rehabilitation research, the challenges associated with running RCTs in this population must be acknowledged. In addition to the aforementioned challenges associated with sample size and heterogeneity, there are also barriers associated with recruitment and matching procedures, outcome measurement sensitivity, participant retention, and the incoherence between statistical significance and clinical relevance.^50^ Despite these challenges, future RCTs of FALT in SCI can reasonably minimize risk of bias in several ways, including: ensuring a strict randomization process; blinding participants and assessors; selecting suitable control conditions; prespecifying primary outcome measures; conducting appropriate statistical analyses; and reporting methods and results in detail.

Running high quality quasi-experimental studies can help to address these challenges,^50^ in addition to increasing the use of pragmatic RCTs, which are more inclusive and reflective of clinical practice, and therefore more generalizable.^55^ Even in a well-designed study, intervention-based research has inherent challenges. Interventions are often long in duration, increasing the likelihood of attrition, and the interventions themselves are often inadequately described, limiting reproducability.^54^ Finding an appropriate, equivalent control group can be especially difficult in this field, given the complex nature of many treatments.^54^ FALT is susceptible to these obstacles, as it requires a large time commitment from participants, and participant blinding and sham-controlled interventions are difficult to achieve due to the stimulation sensation. Continued discussions on the most appropriate study designs to determine the effectiveness of interventions such as FALT and how to mitigate risk of bias in these studies are warranted.

A recent umbrella review examined the use of FES alone or as an adjunct treatment to improve walking in individuals with upper motor neuron impairments.^56^ The authors reported both orthotic and therapeutic effects of FES, but acknowledged that the certainty of the evidence was low to moderate due to similar challenges presented in this review.^56^ Included reviews came to differing conclusions, with 2 SCI-specific reviews describing orthotic effects, 2 showing therapeutic effects, and 1 demonstrating both effects.^56^ Findings were similar for individuals who had experienced a stroke, with some reviews finding therapeutic effects of FES on walking speed and endurance when it was used in combination with other types of rehabilitation, while others reported no effect.^56^ Specific to FALT, a systematic review of FES combined with BWSTT compared to BWSTT alone post stroke found significant improvements on various functional outcome measures, including walking speed.^57^ These findings are inconsistent to the results of this meta-analyses, where FALT was not found to have any significant effects on walking speed or endurance in people with incomplete SCI. Variable conclusions across interventions and populations warrants further investigation with standardized approaches.

## Limitations

This review has some notable methodological limitations. First, the review examined the therapeutic effectiveness of FES combined with any type of locomotor training, such that studies differed in terms of the specific intervention (eg, overground, treadmill, combination therapy). It is possible that results would be different between specific interventions, but there were not enough data to analyze by intervention subtype. Second, there was considerable variability in the training dose across studies. The observed treatment effects may be influenced by differences in total dose rather than the addition of FES alone, and the training dose required to improve walking outcomes cannot be ascertained. Third, the control groups were highly variable, ranging from no intervention to locomotor training without FES to other types of exercise, making it difficult to interpret the true impact of the intervention. Fourth, this review included only a small number of studies, which were often performed in the pilot stage and thus had small sample sizes. For the meta-analysis, only 3 studies were suitable for inclusion.

## Conclusions

This review did not find significant evidence that FALT improves walking speed or endurance for people with motor incomplete SCI relative to other types of locomotor training, although these findings were likely impacted by heterogeneity, limited reporting, and risk of bias in the available research Future trials of FALT for SCI should aim to better standardize and report training dose and stimulation parameters to improve comparability across studies. As FALT demonstrates overall improvements in walking outcomes, it should still be considered a valuable intervention in SCI rehabilitation, especially when other types of locomotor training (eg, RALT or BWSTT) may not be available.

## Supplemental Material

sj-docx-1-nnr-10.1177_15459683251395722 – Supplemental material for Effectiveness of Functional Electrical Stimulation Assisted Locomotor Training on walking Outcomes Following Incomplete Spinal Cord Injury: Systematic Review and Meta-Analysis Supplemental material, sj-docx-1-nnr-10.1177_15459683251395722 for Effectiveness of Functional Electrical Stimulation Assisted Locomotor Training on walking Outcomes Following Incomplete Spinal Cord Injury: Systematic Review and Meta-Analysis by Janelle Unger, Joshua C. Wiener, Prachi Patel, Usman Shakir and Janice J. Eng in Neurorehabilitation and Neural Repair
